# Cardioprotective O-GlcNAc signaling is elevated in murine female hearts *via* enhanced O-GlcNAc transferase activity

**DOI:** 10.1016/j.jbc.2023.105447

**Published:** 2023-11-08

**Authors:** Bhargavi Narayanan, Prithvi Sinha, Roger Henry, Russell A. Reeves, Nazareno Paolocci, Mark J. Kohr, Natasha E. Zachara

**Affiliations:** 1 The Department of Biological Chemistry at the Johns Hopkins University School of Medicine, Baltimore, Maryland, USA; 2 The Department of Environmental Health and Engineering, The Johns Hopkins Bloomberg School of Public Health, Baltimore, Maryland, USA; 3 Division of Cardiology, Department of Medicine, Johns Hopkins University School of Medicine, Baltimore, Maryland, USA; 4 Department of Biomedical Sciences, University of Padova, Padua, Italy; 5 The Department of Oncology at the Johns Hopkins University School of Medicine, Baltimore, Maryland, USA

**Keywords:** O-GlcNAc, glycosylation, cardiac, sex differences, O-GlcNAc transferase, O-GlcNAcase, hexosamine biosynthetic pathway

## Abstract

The post-translational modification of intracellular proteins by O-linked β-GlcNAc (O-GlcNAc) has emerged as a critical regulator of cardiac function. Enhanced O-GlcNAcylation activates cytoprotective pathways in cardiac models of ischemia–reperfusion (I/R) injury; however, the mechanisms underpinning O-GlcNAc cycling in response to I/R injury have not been comprehensively assessed. The cycling of O-GlcNAc is regulated by the collective efforts of two enzymes: O-GlcNAc transferase (OGT) and O-GlcNAcase (OGA), which catalyze the addition and hydrolysis of O-GlcNAc, respectively. It has previously been shown that baseline heart physiology and pathophysiology are impacted by sex. Here, we hypothesized that sex differences in molecular signaling may target protein O-GlcNAcylation both basally and in ischemic hearts. To address this question, we subjected male and female WT murine hearts to *ex vivo* ischemia or I/R injury. We assessed hearts for protein O-GlcNAcylation, abundance of OGT, OGA, and glutamine:fructose-6-phosphate aminotransferase (GFAT2), activity of OGT and OGA, and UDP-GlcNAc levels. Our data demonstrate elevated O-GlcNAcylation in female hearts both basally and during ischemia. We show that OGT activity was enhanced in female hearts in all treatments, suggesting a mechanism for these observations. Furthermore, we found that ischemia led to reduced O-GlcNAcylation and OGT-specific activity. Our findings provide a foundation for understanding molecular mechanisms that regulate O-GlcNAcylation in the heart and highlight the importance of sex as a significant factor when assessing key regulatory events that control O-GlcNAc cycling. These data suggest the intriguing possibility that elevated O-GlcNAcylation in females contributes to reduced ischemic susceptibility.

Cardiovascular disease (CVD), including ischemia–reperfusion (I/R) injury, is impacted by both sex and gender. Sex refers to biological attributes encoded by DNA (*e.g.*, reproductive organs), whereas gender is multidimensional and relates to social and cultural behaviors (1). While gender impacts access to health care and implementation of prevention, sex affects disease presentation, pathophysiology, and treatment response. The prevalence of myocardial infarction (MI; I/R injury) was 4.5% for males and 2.1% for females (>20 years of age) from 2017 to 2020 (2). The risk of MI rises sharply in female’s postmenopause and in young women who have had an oophorectomy (3, 4, 5). In rodent studies, females exhibit enhanced recovery and smaller infarcts compared with males and ovariectomized females (6, 7, 8, 9). Loss of cardioprotection in the aforementioned models has been attributed to a decline in estrogen, namely 17β-estradiol. Reinforcing this hypothesis are data from rodents where treatment with 17β-estradiol, or agonists of estrogen receptors, reduces infarct size and fibrosis while improving heart function (8, 10, 11). However, clinical outcomes have not supported a cardioprotective role for estrogen(s). For instance, hormone replacement therapy–based clinical trials failed to exhibit a reduction in heart disease following hormone administration. In fact, some trials exhibited an increased risk of CVD events suggesting a more complicated picture than simply replacing estrogen (12). These inconsistent findings highlight the importance of examining baseline biological differences between male and female hearts that will help elucidate the influence of both components of biological sex (gonadal hormones and genetics) on heart function basally and in response to injury. While molecular processes that regulate cardiac physiology such as protein S-nitrosylation (SNO) have been studied in male and female hearts (6, 13), to date, the impact of sex on cardioprotective signaling mediated by protein O-GlcNAcylation has not been assessed.

O-linked β-O-GlcNAc, is an essential and dynamic post-translational modification of more than 5000 nuclear, mitochondrial, and cytosolic proteins (14, 15, 16). O-GlcNAc modification, or O-GlcNAcylation, of proteins impacts their function and in turn diverse cellular processes that include proteostasis, metabolism, signal transduction, mitochondrial function, epigenetics, and transcriptional regulation (17, 18, 19, 20, 21). The dynamic cycling of protein O-GlcNAcylation is regulated by two enzymes: the O-GlcNAc transferase (OGT) and the O-GlcNAcase (OGA) that catalyze the addition and removal of O-GlcNAc, respectively (22, 23, 24). OGT uses the nucleotide sugar UDP-GlcNAc, which is synthesized by the hexosamine biosynthetic pathway (HBP). The rate-limiting enzyme of the HBP is the glutamine-fructose 6 phosphate amidotransferase (GFAT) (14, 15). Demonstrating the importance of O-GlcNAcylation in both cell function and physiology, deletion of OGT or OGA in mammals is lethal (25, 26, 27).

A rapid, dynamic, and global elevation of O-GlcNAcylation is observed in response to environmental stressors (*e.g.*, heat shock, oxidative, osmotic) as well as physiological models of tissue injury (*e.g.*, ischemic preconditioning [IPC]) (28, 29, 30, 31, 32, 33, 34, 35, 36). Enhancing O-GlcNAc levels in cells before heat stress promotes survival and the induction of heat shock proteins—suggesting that stress-induced cycling of O-GlcNAc leads to cytoprotection (28, 29). Critically, the cytoprotective role of O-GlcNAc is not restricted to cellular models, with several groups demonstrating that acute increases in protein O-GlcNAcylation are cardioprotective (34, 35, 36, 37, 38, 39, 40, 41, 42, 43). For instance, elevating O-GlcNAc levels prior to ischemia or during reperfusion significantly improves heart function (*ex vivo*) and reduces infarct size (*in vivo*) (33, 34, 35, 36, 37, 39). In contrast, inducible deletion of OGT prior to I/R injury (*in vivo*) results in a significant decline in left ventricular function and increased cardiac fibrosis and cardiomyocyte death (44). Supporting a cytoprotective role of O-GlcNAc in the heart, elevating O-GlcNAc levels in cardiac models suppresses all the hallmarks of I/R injury, including reducing reactive oxygen species release and endoplasmic reticulum stress, preserving mitochondrial membrane potential, inhibiting opening of the mitochondrial permeability transition pore, and preventing calcium overload (37, 42, 45, 46, 47). Despite its well-documented role in cardioprotection and CVD, the mechanisms regulating O-GlcNAc cycling in the heart are ill defined.

To address these gaps in knowledge, we have compared O-GlcNAc cycling in male and female hearts subjected to control, ischemic, and I/R injury *ex vivo*, using methodology that enables measurement of enzymatic activity, protein abundance (OGT, OGA, and GFAT2), and UDP-GlcNAc levels from a single heart. In ischemic samples, lower levels of O-GlcNAc were detected, and this was associated with a reduction in OGT activity. Notably, we observed elevated O-GlcNAcylation in female hearts before and after injury, which was associated with enhanced OGT activity. Collectively, our data and that of others underpin the importance of examining sex differences with the intact influence of hormones on fundamental biochemical signaling pathways that impact cardiac homeostasis and disease.

## Results

### O-GlcNAc levels are enhanced in female hearts compared with age-matched male hearts

**Figure 1:**
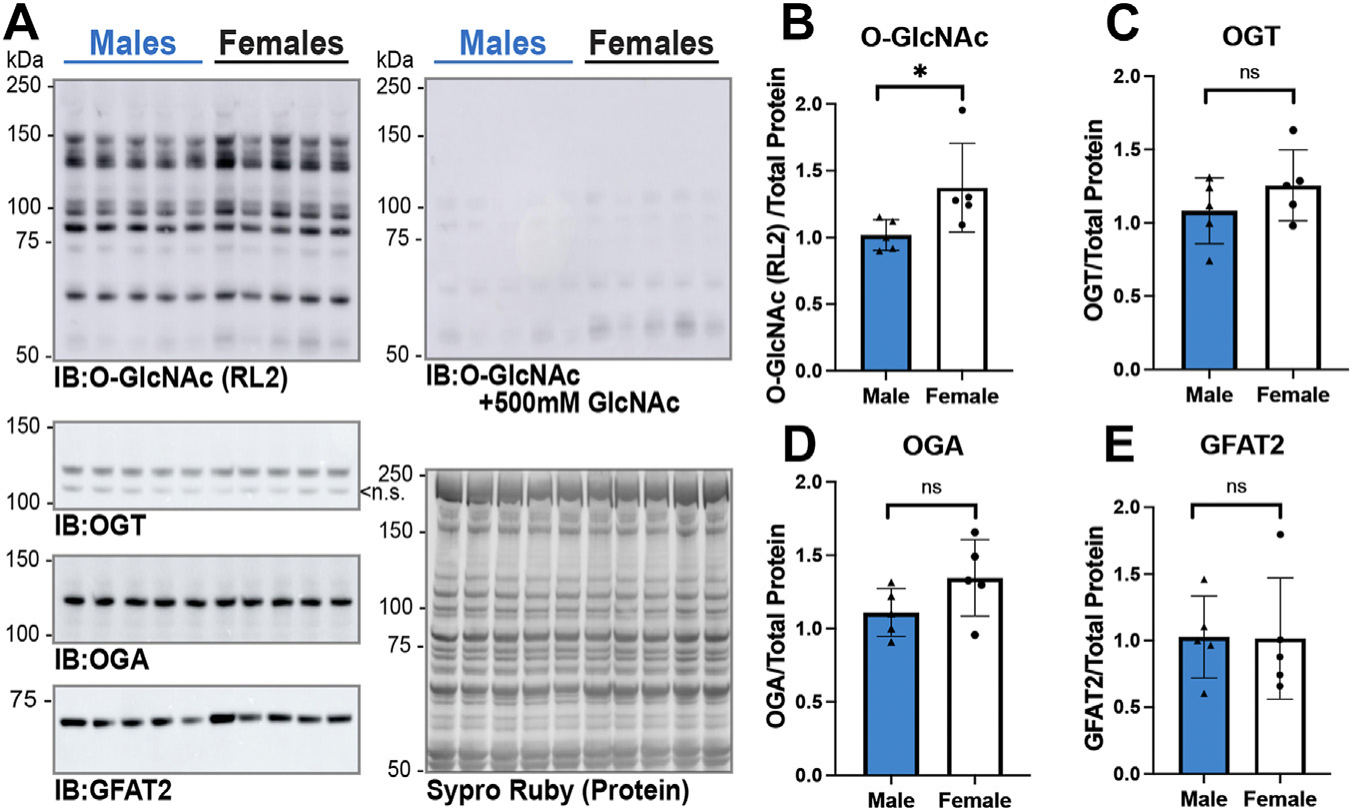
**O-GlcNAc levels are enhanced in female hearts compared with age-matched male hearts.***A*, total tissue lysates of hearts from C57B6/J WT mice (14–18 weeks, N = 5 per sex) were generated using 9 M urea. Proteins were separated by SDS-PAGE (15 or 22.5 μg), electroblotted to nitrocellulose, and the following were detected and quantified: O-GlcNAc (RL2), O-GlcNAc specificity control (RL2 with 500 mM GlcNAc), O-GlcNAc transferase (OGT), O-GlcNAcase (OGA), and glutamine fructose-6-phosphate amidotransferase 2 (GFAT2). Protein load was assessed by total protein membrane stain (SYPRO Ruby). The following signals were quantified and normalized to total protein (*B*) O-GlcNAc (RL2); (*C*) OGT; (*D*) OGA; and (*E*) GFAT2. Statistical test: unpaired Student’s *t* test. ∗*p* < 0.05. *B*–*E*, data are represented as mean ± SD. ns, nonspecific band; O-GlcNAc, O-linked β-GlcNAc.

Motivating our interest in assessing the impact of sex on O-GlcNAcylation are reports demonstrating that *Ogt* is X-linked, that male embryos have lower levels of O-GlcNAc than females in a placental stress model, and that in hearts subjected to transverse aortic constriction (TAC)—OGT abundance is transiently higher in female hearts than in males (27, 48, 49). To characterize O-GlcNAc cycling in male and female murine hearts basally, we assessed the levels of O-GlcNAc as well as the abundance of OGT, OGA, and GFAT2 in total tissue lysates (9 M urea, N = 5/sex) using immunoblotting. We used the RL2 antibody to detect O-GlcNAc, as we have previously shown that this detects cardiac contractile proteins with greater sensitivity than CTD110.6 (50). The data presented in Figure 1 demonstrate elevated O-GlcNAc levels (∼35%) in female hearts compared with age-matched male hearts. As a control, the O-GlcNAc antibody RL2 was competed away with free GlcNAc. The residual signal is due to endogenous immunoglobulin (immunoglobulin G [IgG] heavy chain ∼55 kDa) and has not been included in the quantitation. No significant change in the abundance of OGT, OGA, or GFAT2 was detected. GFAT2 has been reported as the primary myocardial variant of GFAT, and consistent with this report, we were unable to detect GFAT1 in murine heart lysates (*data not shown*). Of note, anti-OGT (O6264; MilliporeSigma) detected two OGT bands; one of which has a similar migration to the mitochondrial isoform of OGT. However, this band was not detected using two antibodies (O6139 and O6014; MilliporeSigma) with independent epitopes (amino acids 833–849 [O6139]; 1024 to 1037 [O6014]; OGT numbering: UniProtKB: O15294-1) present in both full-length and mitochondrial OGT isoforms (*data not shown*). Thus, we conclude that this band is nonspecific. Together, these data demonstrate that elevated levels of O-GlcNAc in female hearts do not arise from changes in the abundance of the O-GlcNAc cycling enzymes (OGT/OGA) or the rate-liming enzyme of the HBP (GFAT2).

### Female hearts begin ischemic contracture and reach hypercontracture earlier than age-matched male hearts

Prior experiments have demonstrated dynamic changes in O-GlcNAcylation in response to ischemia and I/R injury (32, 33, 34, 37, 51). In an *ex vivo* perfusion model in rats, O-GlcNAcylation increases after 10 min of ischemia but declines between 10 and 30 min of ischemia and remains depressed at 60 min of reperfusion (34). To assess the impact of ischemia and I/R injury in mice, we used the Langendorff perfused heart model to collect hearts exposed to either 20 min of ischemia or ischemia with 40 to 120 min of reperfusion. Consistent with data in rat hearts, our pilot studies demonstrate a reduction in O-GlcNAc (CTD110.6) on soluble heart proteins at 20 min of ischemia in male hearts (Fig. S1*A*). We assessed O-GlcNAc at 40, 80, and 120 min of reperfusion, determining that O-GlcNAcylation remains low at 40 and 80 min, before rebounding at 120 min (Fig. S1*B*).

**Figure 2:**
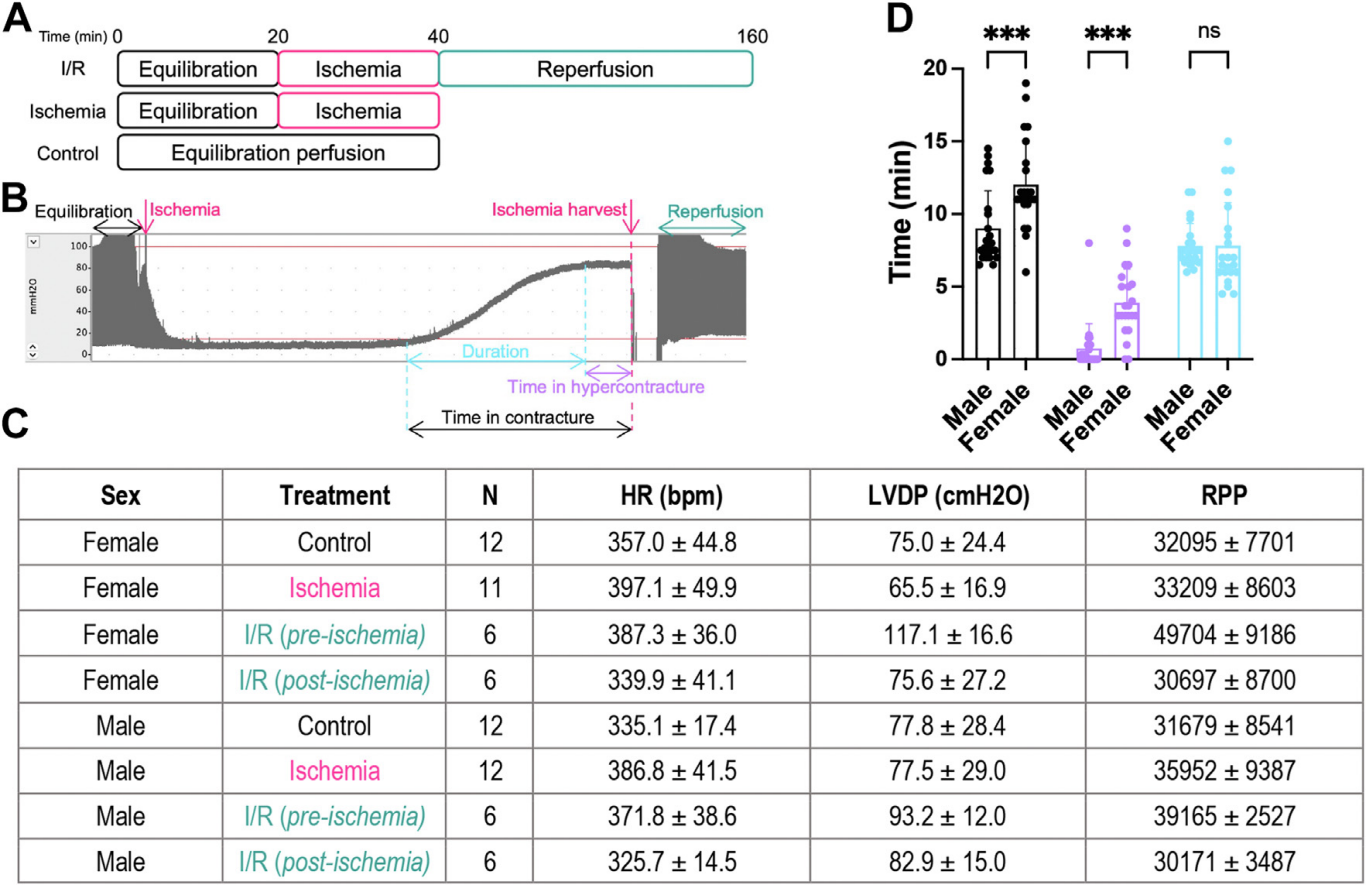
**Female hearts begin ischemic contracture and reach hypercontracture earlier than age-matched male hearts.** Hearts from C57B6/J WT mice (15–21 weeks, N = 6–12 per sex per treatment) were treated *ex vivo* on the Langendorff apparatus. *A*, perfusion protocol: control—40 min perfusion; ischemia—20 min perfusion, 20 min—no-flow ischemia; ischemia–reperfusion (I/R) injury—20 min perfusion, 20 min ischemia, 120 min reperfusion. *B*, representative I/R left ventricular developed pressure (LVDP) trace obtained during perfusion. Critical time points are highlighted. *C*, summary of preischemic and postischemic contractile parameters. Heart rate (HR), LVDP, and rate pressure product (RPP) were recorded for all replicates and represented as mean ± SD. *D*, comparison of time in contracture (•), time in hypercontracture (), and the duration of contracture () between males and females. Statistical test: two-way ANOVA. ∗∗∗*p* < 0.001. Data are represented as mean ± SD.

To determine if sex impacts O-GlcNAcylation and heart function during ischemia and reperfusion, we collected a cohort of murine male and female hearts exposed to 20 min of ischemia (ischemia) or 20 min of ischemia and 120 min of reperfusion (I/R) *ex vivo* on the Langendorff apparatus (Fig. 2*A*). A representative left ventricular developed pressure (LVDP) trace is presented (Fig. 2*B*). Consistent with prior data, our measurements indicated no differences in LVDP and heart rate (HR) between male and female hearts (Fig. 2*C*) (13). Using the pressure traces, we also assessed time in ischemic contracture between male and female hearts (Fig. 2, *B* and *D*). While no difference in the duration of contracture was observed, we detected a sex-dependent difference in the initiation of contracture and the time to hypercontracture (Fig. 2*D*). Female hearts begin to go into contracture and reach hypercontracture faster than male counterparts, possibly as these hearts contain less glycogen content and exhaust energy reserves faster than male hearts (52).

### O-GlcNAc levels cycle in response to I/R injury with little impact on OGT and OGA abundance

**Figure 3:**
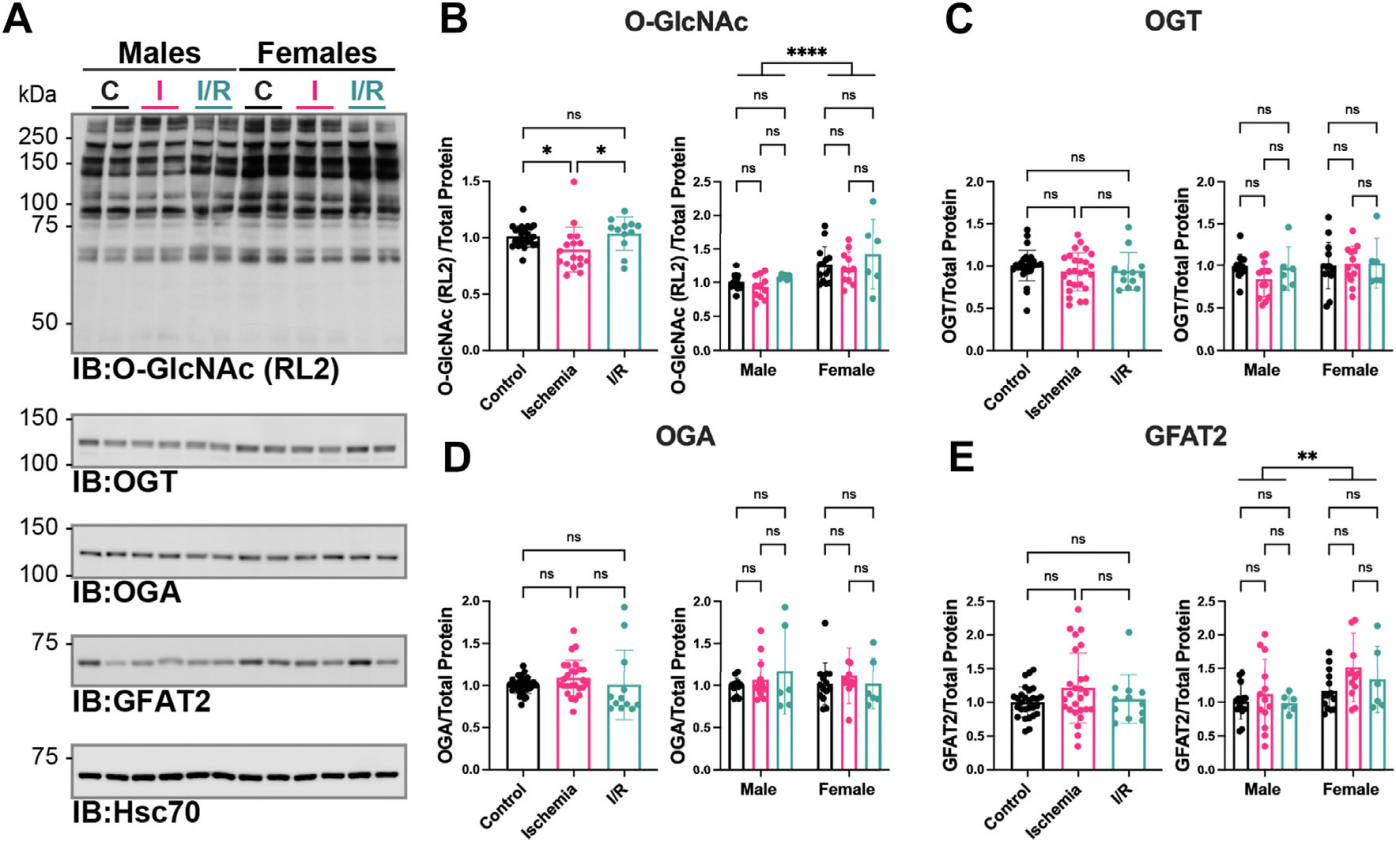
**O-GlcNAc levelscycle in response to I/R injury with little impact on OGT and OGA abundance.***A*, hearts from C57B6/J WT mice (15–21 weeks, N = 6–12 per sex per treatment) were perfused *ex vivo* on the Langendorff apparatus (control [•]; ischemia 20 min []; I/R 20 min ischemia/120 min reperfusion []). Hearts were extracted in 1% N-P40 buffer, and proteins were separated by SDS-PAGE (15 or 22.5 μg), electroblotted to nitrocellulose, and the following were detected: OGT, OGA, GFAT2, heat shock cognate 70 (Hsc70), O-GlcNAc (RL2), and O-GlcNAc specificity control (RL2 with 500 mM GlcNAc, *not shown*). Hsc70 was used as a representative immunoblot for protein load. Protein load was assessed by total protein membrane stain (SYPRO Ruby or Direct Blue-71, Fig. S2). The following were quantified and normalized to total protein. (*B*) O-GlcNAc (RL2); (*C*) OGT; (*D*) OGA; and (*E*) GFAT2. *B*–*E*, pooled male and female data are reported on the *left*. Statistical test: one-way ANOVA. ∗*p* < 0.05. Data parsed by sex are reported on the *right*. Statistical test: two-way ANOVA. ∗∗*p* < 0.01; ∗∗∗∗*p* < 0.0001. Data are represented as mean ± SD. GFAT2, glutamine-fructose 6 phosphate amidotransferase 2; IR, I/R, ischemia–reperfusion; OGA, O-GlcNAcase; O-GlcNAc, O-linked β-GlcNAc; OGT, O-GlcNAc transferase.

To assess O-GlcNAc cycling in injured hearts, we measured O-GlcNAc levels, the abundance of OGT, OGA, and GFAT2, OGT and OGA activity, and UDP-GlcNAc levels. O-GlcNAcylation in the aforementioned hearts was determined in total tissue lysates (1% NP-40) by immunoblotting using the RL2 antibody (Fig. 3*A*). Quantitation of O-GlcNAc immunoblots demonstrated a modest decrease in O-GlcNAcylation after 20 min of ischemia in both male and female hearts (Fig. 3, *A* and *B*). After 120 min of reperfusion, O-GlcNAcylation returns to baseline. Like the data presented in Figure 1*B*, elevated O-GlcNAcylation (∼30%) was detected in female hearts. This observation is especially evident in control and ischemic hearts (Fig. 3, *A* and *B*).

Prior studies have correlated changes in O-GlcNAc with the abundance of the enzymes that cycle O-GlcNAc (OGT and OGA) or are critical to UDP-GlcNAc synthesis (GFAT2) in models of I/R injury, IPC, and remote ischemic preconditioning (rIPC) (32, 33, 51). Thus, we assessed OGT, OGA, and GFAT2 abundance in these hearts (Fig. 3, *C*–*E*). We observed no significant injury-induced changes in the abundance of OGT (Fig. 3*C*), OGA (Fig. 3*D*), and GFAT2. However, like O-GlcNAc levels, we observed elevated GFAT2 abundance in female hearts compared with males (Fig. 3*E*). This observation is unlike our prior data in Figure 1*E* and appears to be driven by a modest elevation in GFAT2 in ischemic female hearts.

### OGT activity is elevated in female hearts

**Figure 4:**
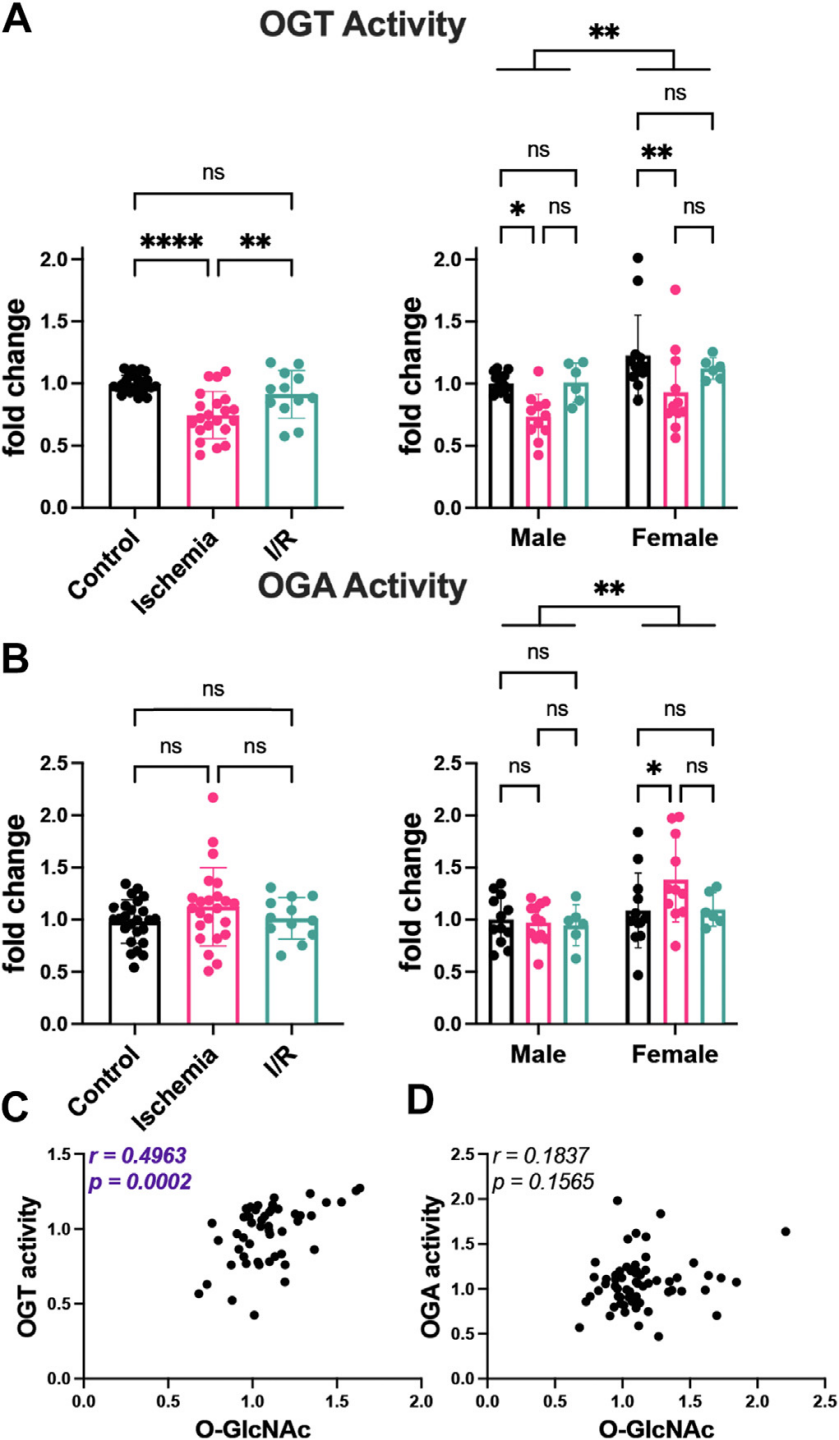
**OGT activity is elevated in female hearts.** Hearts from C57B6/J WT mice (15–21 weeks, N = 6–12 per sex per treatment) were perfused *ex vivo* on the Langendorff apparatus (control [•]; ischemia 20 min []; I/R 20 min ischemia/120 min reperfusion []). Hearts were extracted in 1% NP-40, and equal protein was desalted using a Zebaspin column. *A*, OGT activity was assessed by measuring transfer of ^3^H-GlcNAc to the casein kinase II (CK2) acceptor peptide. *B*, OGA activity was detected using the pseudosubstrate 4-methylumbelliferyl-β-GlcNAc. *A* and *B*, pooled male and female data are reported on the *left*. Statistical test: one-way ANOVA. ∗∗*p* < 0.01; ∗∗∗∗*p* < 0.0001. Data parsed by sex are reported on the *right*. Statistical test: two-way ANOVA. ∗*p* < 0.05; ∗∗*p* < 0.01. Data are represented as mean ± SD. *C*, correlation of OGT activity and O-GlcNAc (RL2). *D*, correlation of OGA activity and O-GlcNAc (RL2). *C* and *D*, *r* = Pearson correlation coefficient. I/R, ischemia–reperfusion; OGA, O-GlcNAcase; OGT, O-GlcNAc transferase.

To provide insight into the mechanisms underpinning sex- and injury-dependent changes in O-GlcNAcylation, we assessed the activity of OGT and OGA in total heart lysates (1% NP-40) using pseudosubstrates as we have previously reported (53, 54, 55). We observed a significant reduction in OGT-specific activity (∼25%) after 20 min of ischemia (Fig. 4*A*) that rebounds during reperfusion. A similar pattern in OGT activity is observed in both males (27%) and females (24%) during ischemia and I/R injury; however, there is a strong sex-dependent impact on OGT activity with female hearts displaying higher OGT activity (∼20%) compared with age-matched male hearts (Fig. 4*A*). While OGA activity appears unaffected by injury in males, ischemia elevates OGA activity in female hearts (27%) (Fig. 4*B*). OGT and OGA activity mirror our pilot studies (Fig. S1), which used soluble lysates from male hearts. As OGT activity and O-GlcNAc showed a similar trend basally and in response to injury in both males and females, we performed a correlation analysis of O-GlcNAc to OGT and OGA activity. While O-GlcNAc did not correlate with OGA activity, O-GlcNAc and OGT activity showed a positive correlation (Fig. 4, *C* and *D*). Collectively, these data suggest that sex-dependent and injury-induced changes to O-GlcNAcylation are driven by changes in OGT activity.

### UDP-GlcNAc levels do not change during injury in male or female hearts

**Figure 5:**
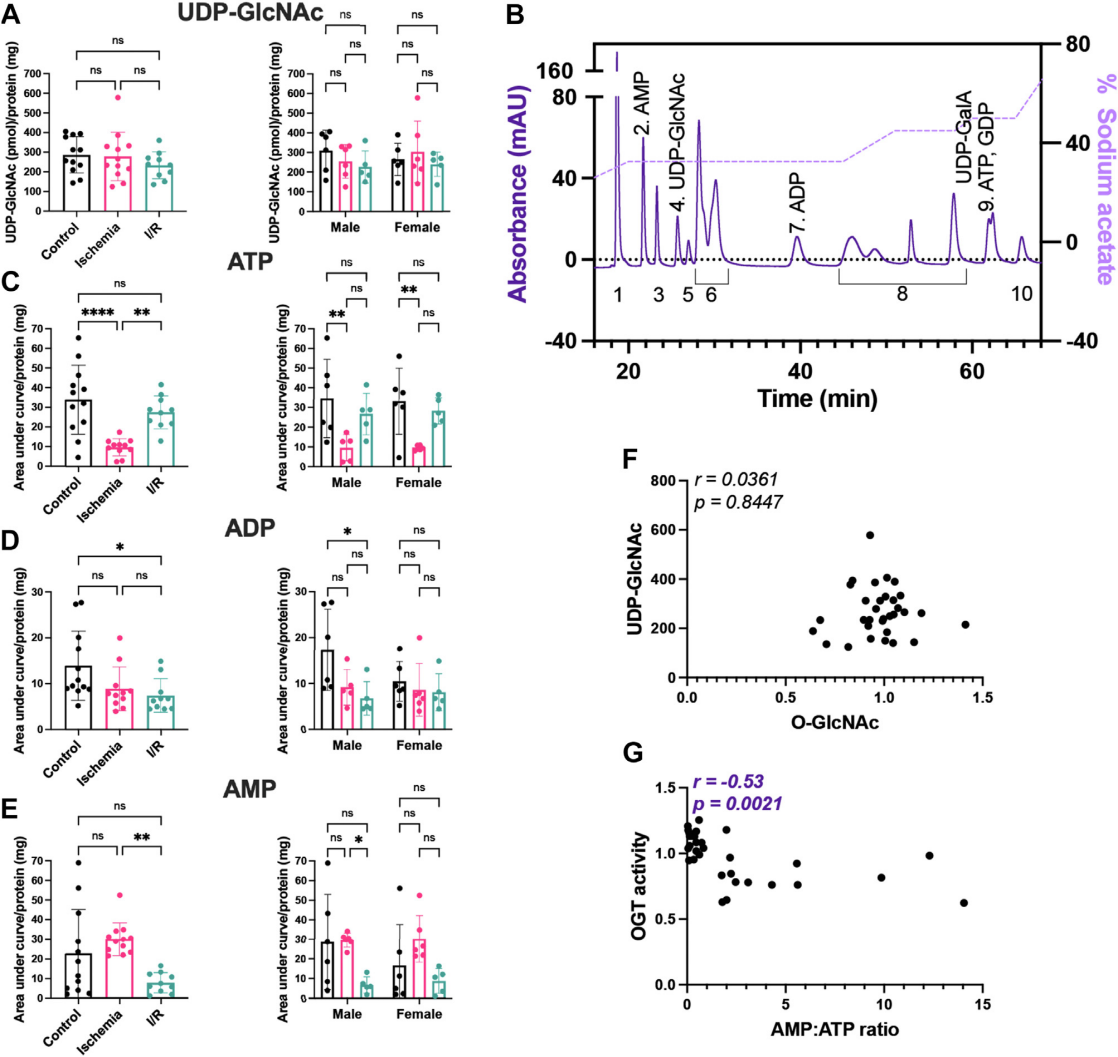
**UDP-GlcNAc levels do not change during injury in both male and female hearts.** Hearts from C57B6/J WT mice (15–21 weeks, N = 5–6 per sex per treatment) were perfused *ex vivo* on the Langendorff apparatus (control [•]; ischemia 20 min []; I/R 20 min ischemia/120 min reperfusion []). Heart nucleotides and nucleotide sugars were extracted in methanol:chloroform, desalted using solid-phase extraction, and analyzed using high-performance anion-exchange chromatography. Nucleotide/nucleotide sugar levels were normalized to total protein: (*A*) UDP-GlcNAc; (*B*) ATP; (*C*) ADP; (*D*) AMP. *A*–*D*, pooled male and female data are reported on the *left*. Statistical test: one-way ANOVA. ∗*p* < 0.05; ∗∗*p* < 0.01; and ∗∗∗∗*p* < 0.0001. Data parsed by sex are reported on the *right*. Statistical test: two-way ANOVA. ∗*p* < 0.05; ∗∗*p* < 0.01. Data are represented as mean ± SD. *E*, representative separation of nucleotide sugars. Peaks are numbered as follows: (1) CMP, CMP-neuraminic acid; (2) AMP; (3) UMP; (4) UDP-GlcNAc; (5) UDP-GalNAc; (6) UDP-glucose, UDP-galactose, UDP-xylose, CDP, GMP; (7) ADP; (8) UDP, GDP-mannose, GDP-fucose, CTP, UTP; (9) ATP, GDP; and (10) UDP-glucuronic acid. The sodium acetate gradient used to achieve nucleotide separation is depicted on the *right Y*-axis. *F*, correlation of UDP-GlcNAc and O-GlcNAc (RL2). *G*, correlation of AMP:ATP ratio and OGT activity. *F* and *G*, *r* = Pearson's correlation coefficient. I/R, ischemia–reperfusion.

Prior studies have indicated that activation of X-box binding protein 1 (Xbp1) by ischemic injury leads to changes in GFAT1 abundance and in turn UDP-GlcNAc levels albeit at latter time points (24 h of reperfusion) (51). As Xbp1 can also be activated by 17-β-estradiol, we assessed UDP-GlcNAc levels in male and female hearts (56). Here, we used a modified Folch extraction to isolate nucleotide sugars, followed by solid phase cleanup, and detection and analysis *via* ion exchange chromatography. We did not observe any sex- or injury-dependent changes to UDP-GlcNAc levels (Fig. 5, *A* and *B*). We note that one previous study in an *ex vivo* perfused rat model of I/R injury detected an elevation of UDP-HexNAc during ischemia that returned to baseline during reperfusion (34). As the method used to detect UDP-GlcNAc was similar, the inconsistency in the UDP-GlcNAc levels between our studies likely results from the method used to normalize nucleotide sugar levels in the heart. We normalized UDP-GlcNAc to total protein instead of wet tissue weight. We chose this approach as ischemic hearts appeared smaller than their counterparts; however, ischemic hearts consistently yielded higher protein (protein yield/wet weight) than control or I/R hearts (Fig. S3*A*). To address the hypothesis that the difference between our observation and those of Fülöp *et al.* arose from differences in normalization technique, we represented our data as UDP-GlcNAc normalized to wet tissue weight (Fig. S3*B*). These data demonstrate an elevation in UDP-GlcNAc during ischemia that returns to baseline during reperfusion. Collectively, these data suggest that normalizing nucleotide sugars to total protein will yield a more accurate assessment of nucleotide sugar levels. In agreement with this conclusion, Olson *et al.* (57) also normalized nucleotide sugars to total protein to avoid variation from including perfusion buffer. While our studies initially solubilized proteins in 1% w/v SDS; further refinement of our approach demonstrates that hydrolyzing the protein in 0.1 M aqueous NaOH results in ∼10% more protein than SDS alone. In future studies, we would suggest directly solubilizing proteins in 0.1 M aqueous NaOH.

In parallel with assessing UDP-GlcNAc, we assessed ATP, ADP, and AMP abundance (Fig. 5, *C*–*E*). While we did not observe any impact of sex on the levels of adenine nucleotides in the heart, as expected, we detected significant changes during injury. After 20 min of ischemia, we detected a 70% decrease in ATP, a 35% decrease in ADP, and a 30% increase in AMP. In contrast, during reperfusion, AMP and ADP levels are depressed, whereas ATP levels return to baseline (Fig. 5, *C*–*E*). Previous data suggest that altered flux through the HBP impacts O-GlcNAc levels; however, we observed no correlation between O-GlcNAc and UDP-GlcNAc levels (Fig. 5*F*). In contrast, we observed a negative correlation between the AMP:ATP ratio and OGT activity (Fig. 5*G*) suggesting that as AMP levels rise, OGT activity is depressed.

## Discussion

O-GlcNAcylation is a critical regulator of cardiac function (40, 43). Prolonged elevation of O-GlcNAc is associated with hypertension, heart failure (pressure overload), and diabetic cardiomyopathy (58, 59, 60). In contrast, IPC and rIPC lead to elevated levels of O-GlcNAc, and augmenting O-GlcNAc transiently is cardioprotective in both *ex vivo* and *in vivo* models of cardiac I/R injury (32, 33, 34, 36, 37, 38, 61). However, the molecular mechanisms by which the heart regulates O-GlcNAcylation are not well understood. In the present study, we document that female hearts have elevated O-GlcNAcylation both basally and during ischemia. As discussed in more detail later, changes in OGT activity drive both sex- and injury-dependent changes in O-GlcNAcylation.

### Elevated O-GlcNAc and OGT activity in female hearts

Our study has identified significantly elevated O-GlcNAcylation in female hearts both basally and in models of ischemia and I/R injury (Figs. 1*B* and 3*B*). While we did not observe any change in the abundance of OGT, OGA, and GFAT2, or UDP-GlcNAc levels, the specific activity of OGT was elevated in female hearts (Fig. 4*A*). *Ogt* is X-linked; however, as protein abundance did not change, these data suggest that sex-dependent signaling regulates the activity of OGT. Our observations are unlike previous data in a murine model of pressure overload–induced cardiac hypertrophy (TAC), where Zhu *et al.* (48) demonstrated no basal differences in cardiac O-GlcNAcylation between males and females. The discrepancy in our datasets can be explained by differences in the O-GlcNAc detection technique. Zhu *et al*. (48) used either RL2 or CTD110.6 to detect O-GlcNAc. In contrast, we used RL2. Our recent studies demonstrate that CTD110.6 has a strong preference for soluble proteins, whereas RL2 will detect both soluble and contractile proteins of the heart (50). Suggesting that females have an enhanced rate of O-GlcNAc cycling compared with males, we also detected elevated OGA activity in females exposed to ischemia (Fig. 4*B*). These data echo studies focused on the cardioprotective role of nitric oxide (NO) and SNO. Female hearts display elevated NO synthase and protein SNO compared with males (6). Counterintuitively, females exhibited higher activity of the enzyme that mediates SNO catabolism, S-nitrosoglutathione reductase. Notably, loss of S-nitrosoglutathione reductase activity promotes protection in male hearts but exacerbates I/R injury in females (13).

The heart is not the only model in which sex-dependent O-GlcNAcylation has been studied. Huynh *et al.* (62) demonstrated that inhibition of OGA differentially associated with sex-dependent changes in mitochondrial parameters as well as the abundance of autophagy-related and neurodegenerative disease–related proteins. However, no difference in O-GlcNAcylation, OGT and OGA abundance, and OGA activity was detected between males and females. In contrast, in the uterus, elevated levels of OGT and O-GlcNAc are detected in the placenta of female embryos when compared with that of males (49). These data suggest that OGT activity and abundance is regulated by different mechanisms in different tissues and/or different times of development. Reinforcing this supposition are data describing the differential regulation of *Ogt* and *Oga* mRNA maturation in cancer models and a murine model in which OGA activity has been ablated. *Ogt* and *Oga* mRNA maturation is controlled by detained intron splicing that in turn responds to cellular O-GlcNAcylation. Thus, when O-GlcNAc levels are high—maturation of *Ogt* mRNA is inhibited and *Oga* mRNA is favored (63). In some cancer models, this regulation appears to be lost, and, as a result, high O-GlcNAc, OGT, and HBP enzyme abundance are observed (64, 65, 66). Similarly, in murine embryos, loss of OGA catalytic activity showed tissue-specific regulation of *Ogt* and *Oga* mRNA abundance and maturation (67). While elevated O-GlcNAcylation and OGA protein abundance were observed in the brain and liver, enhanced maturation of *Oga* mRNA occurred only in the liver, whereas mature *Oga* mRNA levels in the brain remained unchanged. Conversely, mature *Ogt* mRNA levels and protein abundance were found to be reduced only in the brain (67).

### OGT activity and O-GlcNAc are depressed during ischemia

Several studies have reported dynamic cycling of O-GlcNAcylation in the heart during injury. In agreement with prior studies (34), we demonstrate a reduction in O-GlcNAc during ischemia, which rebounds during reperfusion (Figs. S1 and 3*B*). This phenotype is more pronounced in soluble heart lysates (Fig. S1); however, as a significant proportion of the OGT remains associated with the contractile fraction using this lysis method, enzyme assays used total tissue lysates (50). To begin to understand the mechanisms regulating O-GlcNAc cycling, we examined the abundance of the O-GlcNAc-cycling enzymes (OGT, OGA, and GFAT2), OGT and OGA activity, and UDP-GlcNAc levels in hearts, either after 20 min of ischemia or after 120 min of reperfusion. While we do not observe changes in the abundance of OGT, our studies revealed a significant decrease in OGT activity (25%) during ischemia that rebounds during reperfusion (Figs. 3, 4*A*, and S1*A*). Prior studies highlight the physiological significance of modest changes in O-GlcNAcylation. For instance, increased O-GlcNAcylation (20%) in the adipose and muscle of mice results in insulin resistance and hyperleptinemia (68). In the murine heart, adenoviral overexpression of OGT results in a 20% increase in O-GlcNAcylation, as well as cardiac remodeling and dysfunction, increased reactive oxygen species, and altered phosphoinositide 3-kinase signaling (69). Finally, in an inducible cardiomyocyte knockout of OGT, left ventricular dysfunction was exacerbated post-MI; however, O-GlcNAcylation was only depressed by 20% (70).

During ischemia, contractile force declines within 10 s and is abolished within a few minutes. After 10 to 20 min, diastolic tension gradually rises, and ischemic contracture occurs (Fig. 2*B*). While the exact mechanisms underlying contracture are unknown, several studies suggest that the rise in resting tension is mediated by the formation of rigor bridges because of low ATP concentration at myofilaments and changes in cellular Ca^2+^ homeostasis. From our LVDP measurements, we observed that female hearts begin to go into contracture and reach a hypercontracted state faster than male hearts. Consequently, female hearts were in hypercontracture longer at the time of harvest. A similar observation was made by Penna *et al.* (71) using an *ex vivo* perfusion of rat hearts. As female hearts have higher O-GlcNAc basally and during injury, we performed a correlation analysis that showed no positive correlation between O-GlcNAc and time in contracture/hypercontracture (Fig. S4, *A* and *B*). Similarly, no correlation was detected between time in contracture or hypercontracture and OGT activity (Fig. S4, *C* and *D*). We did detect a negative correlation between the AMP:ATP ratio and OGT activity consistent with a reduction in OGT activity during ischemia (Fig. 5*G*). OGT is regulated by kinases that respond to energy balance, such as AMP-activated protein kinase (72, 73) and glycogen synthase kinase 3β (GSK3β) (74). AMP-activated protein kinase activates OGT in a model of glucose deprivation in skeletal muscle myotubes resulting in the increased O-GlcNAcylation of nuclear proteins (73). GSK3β phosphorylates and activates OGT from murine brain extracts (74). Alternatively, Chang *et al.* (75) demonstrated that activation of PKA by forskolin in a model of glucose deprivation decreased O-GlcNAcylation in rat kidney cells. Our data would be consistent with reduced GSK3β and/or enhanced PKA signaling that have been reported during ischemia (76, 77, 78).

As previous studies have implicated HBP flux in regulating O-GlcNAc levels in models of I/R injury (34, 51), our studies addressed the abundance of GFAT2—the rate-limiting enzyme of the HBP. We note that prior studies have demonstrated that both GFAT1 and GFAT2 are expressed in the myocardium; however, GFAT2 is both the more abundant isoform and the primary regulator of cardiac O-GlcNAcylation (79). In agreement with these observations, we were unable to detect GFAT1. We observed little change in GFAT2 abundance, except for female hearts subjected to ischemia (Fig. 3*E*). Nonetheless, we did not detect any changes to UDP-GlcNAc during injury in both males and females (Fig. 5*A*). In a recent study, Olson *et al.* (57) assessed glucose flux through the HBP in an *ex vivo* perfused heart by radiolabeling followed by LC–MS. They demonstrated that reducing cardiac workload by arresting ventricular contractions did not impact HBP flux, and UDP-GlcNAc remained stable from 20 to 40 min of perfusion. These observations agree with our data where we observed no changes to UDP-GlcNAc levels after 20 min of ischemia or after 120 min of reperfusion. Collectively, as no change in UDP-GlcNAc levels were detected, these data suggest that alterations in OGT activity are the major driver of changes in O-GlcNAcylation in the heart and that during early time points in reperfusion, cellular UDP-GlcNAc pools and HBP flux are sufficient to supply OGT.

The role of HBP and protein O-GlcNAcylation on cardiovascular function is well established. Several groups have demonstrated that acute changes in O-GlcNAcylation are cardioprotective, whereas prolonged enhancement or depression promotes heart failure (32, 33, 34, 35, 36, 37, 38, 39, 44, 61, 69, 80, 81, 82, 83, 84, 85, 86, 87, 88). Recent studies have highlighted some mechanisms underlying changes in O-GlcNAcylation during acute stress such as IPC and rIPC and in more chronic settings, such as diabetes, hypertension, cardiac hypertrophy, and MI. In murine and rat pressure overload–induced hypertrophy (TAC) models, elevated O-GlcNAcylation (12–60%) was associated with increased abundance of OGT (20–150%) and GFAT2 (10%). The impact of TAC on OGA abundance is mixed, with both an increase (300%) and decrease (−40%) observed (44, 58, 88). In an MI-induced heart failure model, increased O-GlcNAcylation (60%) was associated with lower abundance of OGA (25%), increased abundance of OGT (110%), and upregulation of miRNA-539 (89). While OGT and OGA activity have not been examined during heart failure, in models such as IPC and rIPC, elevated O-GlcNAcylation (50%) is associated with increased OGT abundance (100%) and activity (100–300%) (32, 33). These data are consistent with our data where changes in O-GlcNAcylation are driven by changes in OGT activity. We posit that transient cycling of O-GlcNAcylation during acute stress targets enzyme activity, whereas prolonged changes in O-GlcNAcylation during sustained cardiac stress likely result from regulation of enzyme abundance. Consistent with this hypothesis, in a murine model of *in vivo* I/R injury, elevated O-GlcNAcylation was observed following 24 h of reperfusion, and this was associated with increased GFAT1 abundance and increased UDP-GlcNAc levels. The upregulation in GFAT1 and HBP flux was mediated by the transcription factor Xbp1-spliced that regulates the unfolded protein response (51).

### Potential mechanisms underpinning sex-dependent O-GlcNAcylation

Sex and gender disparities have been reported for many human diseases, including CVDs. While hormones play an important role in mediating sex differences, biological sex is determined by both gonadal hormones and sex-specific programs controlled genetically (X and Y chromosomes) (90). The impact of the latter was recently highlighted in a study utilizing the Four Core Genotypes model in which the gene *Sry* is deleted from the Y chromosome and inserted into an autosome resulting in the following four genotypes; XX (ovaries), XX_(*Sry*+)_ (testes), XY^−^_(*Sry*+)_ (testes), and XY^−^ (ovaries). Although these mice develop testes, gonadal sex is uncoupled from the sex chromosome complement. Shi *et al.* (90) identified over 500 proteins that segregate with ovaries/testes and ∼150 proteins cosegregating with sex chromosomes in the murine heart. These studies highlighted the glycoprotein A1BG, the abundance of which is elevated in adult and embryonic (prior to gonad formation) female hearts. Loss of A1BG resulted in severely compromised cardiac function only in females (90). Deletion of *Sry* had no impact on the abundance of OGT and OGA in males or females. This finding is consistent with our observations and suggests that regulation of OGT activity is mediated by alternate mechanisms involving the sex chromosomes or through hormone-mediated signaling. Support for the latter comes from reports in skeletal muscle where hormone replacement therapy in postmenopausal women suppressed age-associated upregulation of *Ogt* mRNA and increased *Oga* mRNA levels compared with controls (91). In a separate study in pregnant normotensive and spontaneously hypertensive (primary hypertension model) rats, O-GlcNAcylation was significantly reduced in the thoracic aorta and mesenteric arteries in both groups during pregnancy (92). Estrogen signaling is mediated by the binding of estrogen to the estrogen receptor α, which can then activate multiple signaling pathways that include activation of PI3K/Akt, PKA, mitogen-activated protein kinase, and changes in calcium and NO (93). Notably, OGT is inhibited by nitrosylation. In contrast, denitrosylation of OGT resulted in the upregulation of OGT catalytic activity and increased O-GlcNAcylation (94). However, our observations of elevated O-GlcNAc levels in females are not consistent with enhanced NO in females targeting OGT. Consistent with this observation, OGT was not found in recent screens focused on identifying protein SNO in the heart (6, 95).

To the best of our knowledge, there has been no assessment of the impact of sex or sex hormones (*e.g.*, estrogen) on OGT/OGA abundance, activity, UDP-GlcNAc, and protein O-GlcNAcylation during cardiac I/R injury. We have demonstrated elevated O-GlcNAcylation in female hearts before and after injury that is consistent with the observed elevation in OGT activity in females compared with males. These findings support the hypothesis that the specific activity of OGT is targeted by biological sex (genetics and sex hormones), thereby impacting cardiac O-GlcNAcylation. Further research including, but not limited to, the assessment of the male and female cardiac O-GlcNAcome, and regulation of OGT activity by post-translational modifications or protein interactors, would provide more insight into its sex-dependent implications on the cardiovascular system. Such studies will also promote the development of sex-specific therapeutics for the treatment of CVDs. Collectively, these data highlight the importance of studying O-GlcNAc in both males and females and suggest that sex-dependent changes in O-GlcNAcylation could alter disease risk or resilience.

## Experimental procedures

### Reagents

All chemicals and reagents were of the highest grade and supplied by MilliporeSigma or Thermo Fisher Scientific, unless otherwise indicated.

### Antibodies

The following antibodies were used: anti-OGT (O6264; MilliporeSigma, antimouse immunoglobulin M-horseradish peroxidase (HRP) (catalog no.: A8786; MilliporeSigma), antimouse IgG-HRP (catalog no.: NA931; Cytiva), anti-rabbit IgG-HRP (catalog no.: NA934; Cytiva); anti-GFAT2 (catalog no.: ab240316; Abcam); anti-heat shock cognate (Hsc) 70 (catalog no.: sc-7298; Santa Cruz Biotechnology); and antichicken IgY-HRP (catalog no.: A30-104p; Fortis Life Sciences). Anti-O-GlcNAc (RL2 and CTD110.6) (96, 97), anti-OGT (AL24) (22) and anti-OGA (345) (98) antibodies were shared by Dr Gerald Hart (CCRC, University of Georgia) and antigen (CTD110.6, AL24, 345) or affinity (RL2) purified prior to use.

### Preparation of tissue lysates

The following lysis buffers were used in this study: urea buffer (25 mM Hepes [pH 7.4], 9 M urea), 1% NP-40 buffer (50 mM Tris–Cl [pH 8.0] with 150 mM NaCl and 1% [v/v] Nonidet P-40), or soluble lysis buffer (50 mM Hepes [pH 7.4]) supplemented with the following inhibitors immediately prior to use: protease inhibitor cocktail sets II & III (MilliporeSigma); 10 μM PUGNAc (A7229, MilliporeSigma); 0.1 mM PMSF; 0.5 μM Thiamet G (synthesized by SD ChemMolecules LLC); 10 mM NaF; and 10 mM β-glycerolphosphate. Heart tissue, powdered under liquid nitrogen, was homogenized using a polytron (Cole-Parmer) in the indicated extraction buffer (10 ml/g of heart powder for 1% NP-40 buffer and 20 ml/g of heart powder for urea buffer) for 2 × 30 s. Tissue debris were pelleted at 18,000*g* (1% NP-40: 20 min, 4 °C; urea: 20 min, 21 °C). Protein concentration was assessed using the Pierce 660 nm colorimetric protein assay according to the manufacturer’s instructions (Thermo Fisher Scientific).

### Electrophoresis and Western blotting

Prior to immunoblotting, samples were desalted and delipidated by methanol–chloroform precipitation and resuspended in loading buffer at 1.5 mg/ml (99). Equal protein was separated by SDS-PAGE using Bis–Tris (NuPAGE; Thermo Fisher Scientific) polyacrylamide gels. Proteins were electroblotted to nitrocellulose (0.45 μm, Bio-Rad) and blocked with 3% w/v nonfat milk in 50 mM Tris, pH 7.5, 150 mM NaCl, 0.05% (v/v) Tween-20; 1 h, 21 °C. Membranes were incubated with primary antibodies (overnight, 4 °C) and subsequently with HRP-conjugated secondary antibodies (1 h, 21 °C). Western blots were developed using Immobilon Western chemiluminescent substrate (MilliporeSigma) and captured on a chemiluminescent imaging system (Amersham Biosciences; RGB600 Imager) or using autoradiography film (Amersham Hyperfilm ECL; GE Healthcare). Protein load was assessed by total protein membrane staining (Sypro Ruby [Bio-Rad] or Direct Blue-71 [MilliporeSigma]). Quantitation of Western blots and total protein was performed using Amersham Imager 600 analysis software. A minimum of two biological replicates were assayed in at least four independent experiments. Loading order was changed in independent experiments to avoid positional bias.

### OGA activity assays

Assays were performed as previously described (55). Briefly, lysates at equal concentration (1 mg/ml) were desalted into desalting buffer (20 mM Tris, pH 7.8, 20% [v/v] glycerol) using Zeba spin desalting columns (catalog no.: 89808; Thermo Fisher Scientific), and the protein concentration was reassessed (Pierce 660, *described previously*). The activity of OGA in tissue lysates (10 μg) was measured in duplicate (technical replicates) in black 96-well plates using 1 mM 4-methylumbelliferyl-GlcNAc (MilliporeSigma) in 50 mM sodium cacodylate, pH 6.4, 0.3% (w/v) bovine serum albumin and 100 mM GalNAc (1 h, 37 °C). 100 mM GalNAc is expected to inhibit lysosomal hexosaminidases; however, to rule out contaminating lysosomal hexosaminidase activity, lysates were also assayed in the aforementioned conditions using 1 mM 4-methylumbelliferyl-GalNAc (MilliporeSigma). Assays were quenched with three volumes of glycine, pH 10.75, and the fluorescence intensity was measured using the SpectraMax i3x (Molecular Devices; excitation 368 nm, emission 450 nm). Activity was corrected for protein and expressed as a fold change over the average of male controls. A minimum of two biological replicates were assayed in at least four independent experiments. Loading order was changed in independent experiments to avoid positional bias.

### OGT assays

Assays were performed as previously described (55). Briefly, lysates were desalted as described previously (OGA assays). The activity of OGT in tissue lysates (10 μg) was assessed in triplicate (technical replicates) using 0.5 μCi per replicate of [^3^H]UDP-GlcNAc (American Radiolabeled Chemicals; specific activity 60 Ci/mmol) and 1 mM casein kinase II acceptor peptide (PGGSTPVSSANMM; The Johns Hopkins University School of Medicine Synthesis and Sequencing Facility) in 50 mM sodium cacodylate, pH 6.4, 0.3% (w/v) bovine serum albumin (1 h, 37 °C; 50 μl total assay volume). Assays were quenched with three volumes of 50 mM formate and 500 mM NaCl. Samples were loaded onto a C_18_ reversed phase 96-well plate (Phenomonex; 100 mg resin/well), activated with methanol, and equilibrated in 50 mM formate, 500 mM NaCl. Plates were washed sequentially with 4 ml of 50 mM formate containing 500 mM NaCl, water, and 50 mM formate. The product was eluted from the column with 1 ml of 100% methanol, and the incorporation of radiolabeled GlcNAc was assessed by liquid scintillation counting. Activity was corrected for protein and expressed as a fold change over the average of male controls. A minimum of two biological replicates were assayed in at least four independent experiments. Loading order was changed in independent experiments to avoid positional bias.

### Langendorff heart perfusion and treatment protocol

All animal studies were carried out in accordance with the guidelines of the Johns Hopkins Institutional Animal Care and Use Committee under protocol #MO22M213. Male and female C57BL6/J WT mice (Jackson Laboratory) 15 to 21 weeks of age were used in these studies. Mice were anesthetized with I.P. injection of ketamine and xylazine (50 mg/kg) and anticoagulated with 100 μl of heparin (1000 U) administered into the inferior vena cava. Hearts were rapidly excised and placed in ice-cold Krebs–Henseleit (KH) buffer containing 120 mM NaCl, 11 mM d-glucose, 25 mM NaHCO_3_, 1.75 mM CaCl_2_, 4.7 mM KCl, 1.2 mM MgSO_4_, and 1.2 mM KH_2_PO_4_. The aorta was cannulated and retrogradely perfused under constant pressure of 100 cmH_2_O and temperature of 37 °C with oxygenated (95% O_2_/5% CO_2_) KH buffer. After cannulation, a saran wrap balloon was immediately inserted into the left ventricle to measure HR pressure and HR, LVDP, and HR. Control hearts were perfused for 40 min. For ischemia and I/R hearts, after 20 min of equilibration perfusion, the flow of oxygenated KH buffer through hearts was halted to induce a global normothermic ischemia for 20 min. To induce I/R injury, after 20 min of ischemia, the flow of oxygenated KH buffer was re-established for 120 min of reperfusion. At the end of perfusion, hearts were rapidly frozen in liquid nitrogen and stored in −80 °C. Rate pressure product, the product of LVDP and HR, was calculated and used to measure cardiac contractile function. Only hearts that reached an LVDP of >50 cmH_2_O and an HR of >300 bpm were included. LVDP measurements were also used to assess ischemic contracture in ischemic and I/R hearts. Time in contracture was calculated as the time from which LVDP begins to increase during ischemia until the end of the ischemic period. Time in hypercontracture was calculated as the time from which LVDP reaches its maximum value during ischemia until the end of the ischemic period. Duration was calculated as the time between the beginning of contracture and hypercontracture.

### Extraction of nucleotide sugars

The following extraction buffer was used to extract nucleotide sugars: methanol:water:chloroform (2:0.8:2) containing the internal standard UDP-galacturonic acid (25 μM) that is not found in mammals. Hearts were homogenized in extraction buffer using a hard tissue lysing kit (Precellys CK28 Lysing Kit; Bertin Technologies) in a bead-mill tissue homogenizer for 2 × 30 s cycles at 0 °C, 7200 rpm (Precellys Evolution 24; Bertin Instruments). Proteins were pelleted at 18,000*g* (10 min, 4 °C). Equal amounts of the aqueous layer, which contains the nucleotide sugars, was dried in a SpeedVac. The organic layer was discarded, and the protein pellet was extracted in 50 mM Tris–Cl (pH 7.5), 1% (w/v) SDS, and quantified (bicinchoninic acid protein assay). The remaining protein pellet was hydrolyzed in 0.1 M aqueous NaOH, quantified (bicinchoninic acid protein assay), and the total protein yield was determined. Nucleotide sugars were resuspended in 10 mM ammonium bicarbonate, pH 8.0, and solid-phase cleanup was performed using ENVI-Carb SPE tubes (57109-U, Supelclean; MilliporeSigma) (100). Columns were activated with methanol, followed by 60% (v/v) acetonitrile, 0.3% (v/v) formic acid, and pH 9 (with ammonia). Columns were equilibrated in water prior to the addition of sample and washed with water followed by 60% (v/v) acetonitrile. Nucleotide sugars were eluted using 2 x× 0.5 ml 60% (v/v) acetonitrile, 0.3% (v/v) formic acid, and pH 9 (with ammonia). The elution was dried in a SpeedVac. Nucleotide sugars were resuspended in water, filtered through a 0.22 μm hydrophilic polyvinylidene difluoride membrane (UFC30GVNB; MilliporeSigma), and analyzed by high-performance anion exchange chromatography (HPAEC). A minimum of two biological replicates were assayed in at least four independent experiments.

### Quantitation of UDP-GlcNAc using HPAEC

Nucleotides and nucleotide sugars (25 μl) were injected onto a Dionex CarboPac column (PA20, 3 × 150 mm; catalog no.: 060142, Thermo Fisher Scientific), equipped with a Dionex CarboPac precolumn (PA20, 3 × 30 mm; catalog no.: 060144, Thermo Fisher Scientific), and analyzed on a Dionex ICS-5000^+^ (Thermo Fisher Scientific) running Chromeleon software (version 7.0; Thermo Scientific). The borate-aided HPAEC separation method was adapted from Oikari *et al*. (101). The column was equilibrated in 120 mM borate (pH 7.5) at 400 μl/min at 60 °C. Nucleotide separation was achieved by introducing 1 M sodium acetate (pH 7.0); a linear gradient of 0 to 32.5% between 0 and 20 min, isocratic conditions between 20 and 45 min at 32.5%; a gradient of 32.5 to 45% between 45 and 51 min, isocratic conditions from 51 to 58 min at 45%; a linear gradient of 40 to 50% between 58 and 60 min; isocratic conditions from 60 to 65 min at 50%; and a linear gradient of 50 to 75% between 65 and 70 min. The column was regenerated in 75% sodium acetate (3 min). Nucleotides were detected at an absorbance of 254 nm. The area under the curve was used to quantify nucleotides per milligram of protein.

### Statistical analysis

Graphs and statistical analysis were performed using the GraphPad Prism software (version 9; GraphPad Software, Inc). Data are reported as mean with error bars representing SD. Biological replicates (N) for each experiment are indicated in the figure legends. A two-way unpaired Student’s *t* test was used for the comparison of two groups (Fig. 1). For the comparison of multiple groups or two groups within each treatment, we utilized a one- or two-factor ANOVA as appropriate. An ordinary one-way ANOVA with Tukey’s multiple comparison test was performed with all pooled male and female data to compare between multiple groups (Figure 3, Figure 4, Figure 5, S1, and S3). An ordinary two-way ANOVA with Sidak’s multiple comparison test was used to compare between males and females within each group (Figure 2, Figure 3, Figure 4, Figure 5 and S3). Pearson correlation coefficients were computed for all correlation analyses (Figs. 4, 5, and S4).

## Data availability

A summary of all original data is presented in the article, including all individual data points collected. Unprocessed data are available on request from the corresponding author: Natasha Zachara (nzachara@jhmi.edu).

## Supporting information

This article contains supporting information.

## Conflict of interest

The authors declare that they have no conflicts of interest with the contents of this article.
