# Supplementary material for: Cardioprotective O-GlcNAc signaling is elevated in murine female hearts via enhanced O-GlcNAc transferase activity
Source: J Biol Chem. 2023 Nov 8;299(12):105447. doi: 10.1016/j.jbc.2023.105447 (PMC10711226; doi:10.1016/j.jbc.2023.105447)
Supplement: Supporting information [file mmc1.pdf]

Supplemental Figures

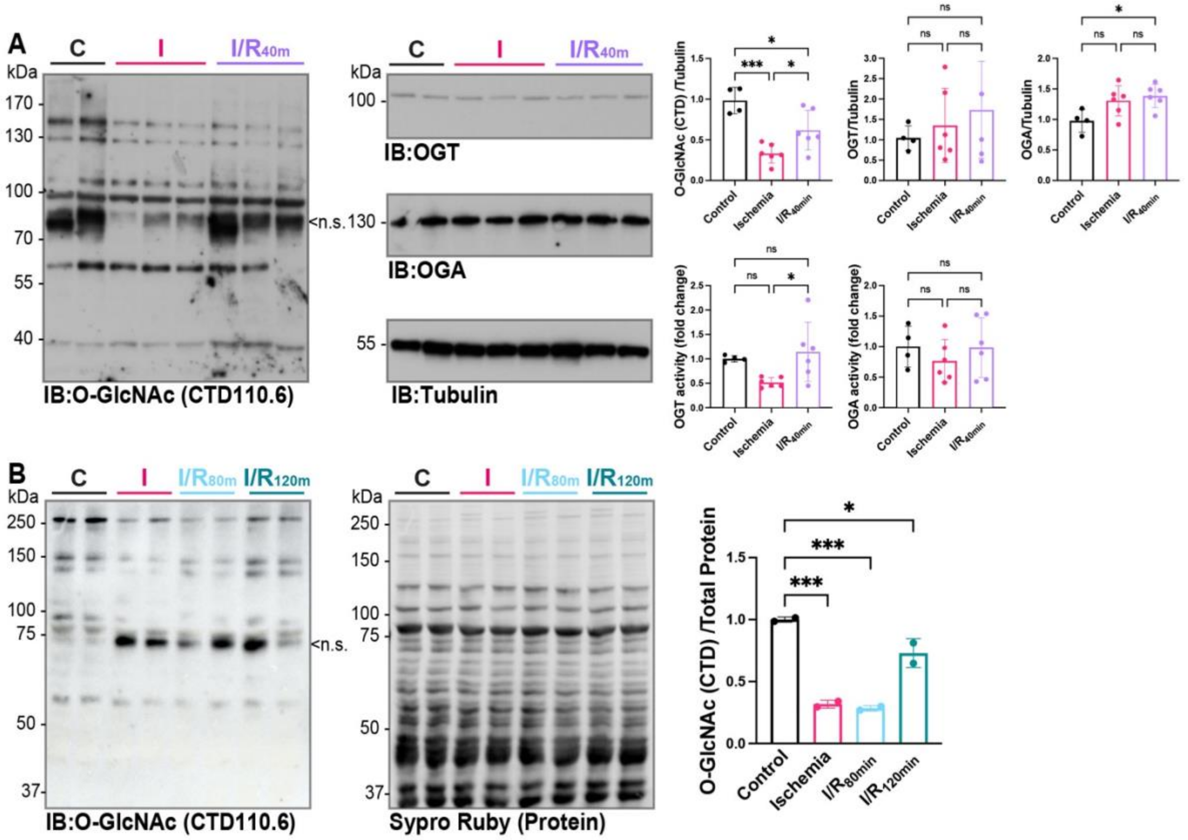

Supplemental Figure 1. O-GlcNAcylation cycles in response to injury in male hearts.

A) Hearts from C57B6/J WT mice (Male, 14 weeks, N=4-6 per treatment) were perfused *ex vivo* on the Langendorff apparatus (control (C); ischemia 20min (I); I/R 20 min ischemia/40 min reperfusion (I/R)). Hearts were extracted in soluble lysis buffer (50mM Hepes, pH 7.4) and proteins were separated by SDS-PAGE (10µg), electroblotted to nitrocellulose, and the following were detected: OGT, OGA, O-GlcNAc (CTD110.6), and Tubulin. n.s. – non-specific band. CTD110.6, OGT, and OGA signal were quantified and normalized to Tubulin. **B)** Hearts from C57B6/J WT mice (Male, 18 weeks, N=2 per treatment) were perfused *ex vivo* on the Langendorff apparatus (control (C); ischemia 20min (I); I/R 20 min ischemia/80 min reperfusion (I/R) or 20 min ischemia/120 min reperfusion (I/R)). Hearts were extracted in 0.5% NP40 buffer (15mM HEPES, pH 7.3 with 250mM NaCl, 10% (v/v) glycerol, and 0.5% (v/v) Nonidet P-40) and proteins were separated by SDS-PAGE (10µg), electroblotted to nitrocellulose, and the following were detected: O-GlcNAc (CTD110.6). n.s. – non-specific band. Protein load was assessed by total protein membrane stain (Sypro Ruby). CTD110.6 signal was quantified and normalized to total protein. **A, B)** Statistical test: 1-way ANOVA. * $p<0.05$; *** $p<0.001$.

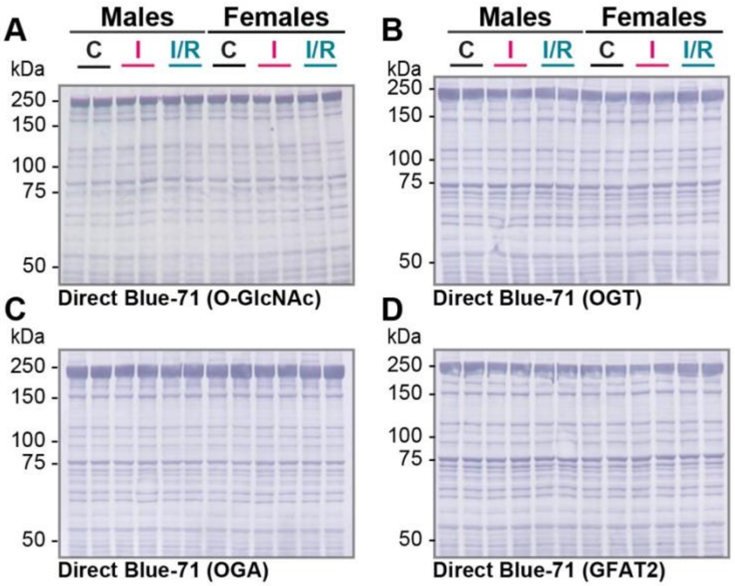

Supplemental Figure 2. Protein load assessed by total protein membrane staining using Direct Blue-71.

Hearts from C57B6/J WT mice (15-21 weeks, N=6-12 per sex per treatment) were perfused *ex vivo* on the Langendorff apparatus (control (C); ischemia 20min (I); I/R 20 min ischemia/120 min reperfusion (I/R)). Hearts were extracted in 1% NP40 buffer and proteins were separated by SDS-PAGE (15 or 22.5µg), and electroblotted to nitrocellulose. Protein load was assessed by total protein membrane stain Direct Blue-71 for each membrane in **Fig. 3**. **A)** O-GlcNAc (RL2); **B)** OGT; **C)** OGA; and **D)** GFAT2.

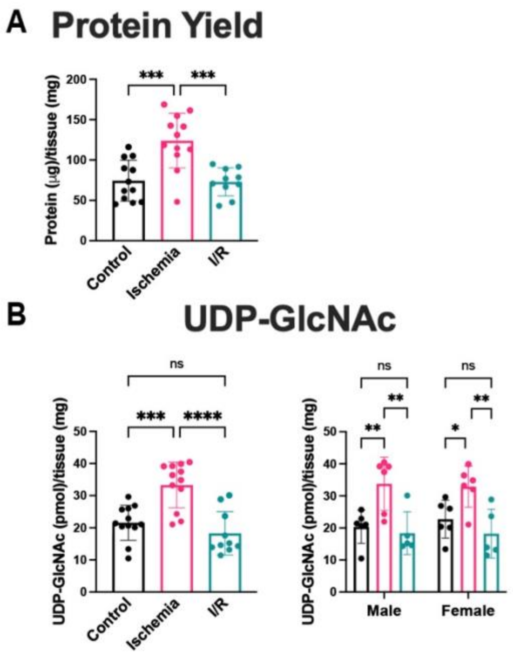

Supplemental Figure 3. UDP-GlcNAc levels normalized to tissue wet weight lead to incorrect representation of data.

Hearts from C57B6/J WT mice (15-21 weeks, N=5-6 per sex per treatment) were perfused *ex vivo* on the Langendorff apparatus (control (•); ischemia 20 min (•); I/R 20 min ischemia/120 min reperfusion (•)). Heart nucleotides and nucleotide sugars were extracted in methanol:chloroform, desalted using solid-phase extraction and analyzed using high performance anion-exchange chromatography. **A)** Total protein yield/mg of tissue wet weight; pooled male and female data are reported. Statistical test: 1-way ANOVA. *** p<0.001. **B)** Nucleotide sugar levels were normalized to tissue wet weight. UDP-GlcNAc; pooled male and female data are reported on the left. Data parsed by sex are reported on the right. Statistical test: 1-way ANOVA. *** p<0.001; **** p<0.0001. Data parsed by sex are reported on the right. Statistical test: 2-way ANOVA. * p<0.05; ** p<0.01. Data is represented as mean ± S.D.

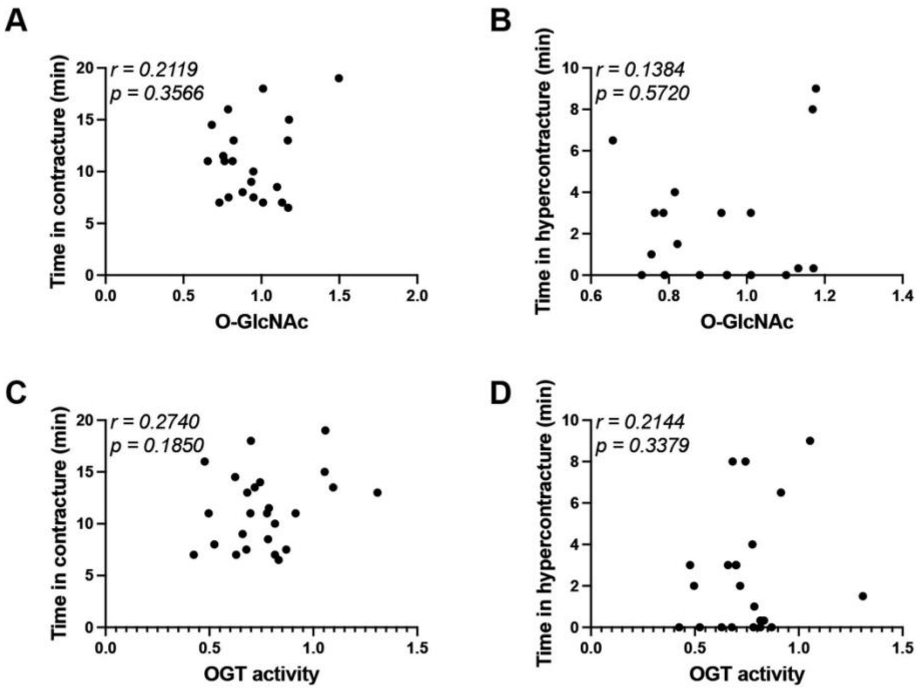

Supplemental Figure 4. O-GlcNAc levels correlate with contracture.

Hearts from C57B6/J WT mice (15-21 weeks, N=6-12 per sex per treatment) were perfused *ex vivo* on the Langendorff apparatus (ischemia 20 min; I/R 20 min ischemia/120 min reperfusion). LVDP was recorded for all samples from start to finish. Time from start of contracture until harvest (time in contracture) and time from full hypercontracture until harvest (time in hypercontracture) was calculated for all ischemic samples from LVDP measurements. **A)** Correlation of O-GlcNAc (RL2) and time in contracture. **B)** Correlation of O-GlcNAc (RL2) and time in hypercontracture. **C)** Correlation of OGT activity and time in contracture. **D)** Correlation of OGT activity and time in hypercontracture. **A-D)** *r* = Pearson correlation coefficient.
